# Mitigation of Volume Changes in Alkali-Activated Slag by Using Metakaolin

**DOI:** 10.3390/ma18112644

**Published:** 2025-06-05

**Authors:** Maïté Lacante, Brice Delsaute, Stéphanie Staquet

**Affiliations:** 1 BATir Department (LGC), Université libre de Bruxelles (ULB), 1050 Brussels, Belgium; maite.lacante@ulb.be (M.L.); brice.delsaute@ulb.be (B.D.); 2 CRIC-OCCN, 1050 Brussels, Belgium

**Keywords:** metakaolin, blast-furnace slag, autogenous strain, coefficient of thermal expansion, compressive strength

## Abstract

This research investigates whether metakaolin can be used as a partial substitution for slag to mitigate significant volume changes in alkali-activated slags. Its effect on compressive strength and workability (as well as on isothermal calorimetry, autogenous strain, and coefficient of thermal expansion (CTE)) were found to depend on both the type and concentration of the alkaline activator. When using 8 M and 10 M sodium hydroxide (NaOH), increasing the substitution rate increased the compressive strength. With sodium silicate (Na_2_SiO_3_), compressive strength decreased as the substitution increased. Isothermal calorimetry revealed metakaolin’s dilution effect at 10% substitution. With 8 M NaOH, a third reaction peak appeared, whose magnitude increased with the substitution rate, while the second peak decreased. The swelling was increased at 10% substitution, followed by constant shrinkage in case of NaOH-activation. Shrinkage was mitigated with Na_2_SiO_3_-activation. Higher substitutions with 8 M NaOH resulted in a significant increase in the shrinkage rate and CTE, occurring when the third reaction peak appeared. A 10% substitution delayed the CTE increase but resulted in higher later-age values (dilution effect). The 20% substitution led to a similar final CTE value at 300 h, while 30% substitution resulted in a decrease in CTE after the initial increase.

## 1. Introduction

Portland cement-based concrete remains the predominant material in the construction industry. Despite many advantages like versatility and fire resistance, the manufacturing of Portland cement (PC) has become a major environmental concern because it accounts for approximately 5 to 8% of the global CO_2_ emissions [1,2,3]. To reduce the ecological footprint of PC, alternative binders such as alkali-activated materials (AAMs) have emerged as promising substitutes [4,5]. These materials offer good thermal resistance and fire resistance while being more ecological and durable. However, one of the key challenges associated with AAMs is their significant volume changes when at an early age [6,7,8], often resulting in the development of internal tensile stresses and subsequent microcracking, especially in restrained conditions. This can be the case when the material is used in mortar and concrete, and especially when it is used in a structure. Investigation into the mitigation possibilities of AAMs is crucial to mitigate volume changes to reduce the cracking potential of these materials in restrained conditions. This highlights the importance of understanding and mitigating early-age volume changes.

These volume changes include autogenous shrinkage and thermal strains (related to a high coefficient of thermal expansion (CTE)). Previously, Lacante et al. [9] investigated the effect of the curing temperature. They revealed that increased curing temperature led to reduced autogenous shrinkage but resulted nevertheless in increased CTE, increasing thermal strains [9]. Another mitigation method is the incorporation of alternative materials. Limestone filler and metakaolin have been shown to reduce these volume changes [10,11]. A previous study investigated a substitution with limestone filler [12]. The present study investigates the substitution of slag with metakaolin.

Metakaolin is a type of calcined clay produced by heating kaolin clay to temperatures between 600 °C and 900 °C. The calcination process can be carried out using two methods. Flash calcination consists in exposing the solid particles of kaolin clay to a stream of gas at 700 °C for a few seconds before quickly cooling them with air cooling cyclones. This process consumes 2.2 MJ/ton of metakaolin, representing an 80% reduction in the energy consumption compared to the production of PC [13,14,15]. The second method is more traditional and consists of a slow calcination by inserting the kaolin clay in a rotary kiln in which the material is heated to high temperature in a continuous process: in this case, the duration of the process takes 3 to 5 h due to the lower heating rates of the device, which operates between 650 °C and 700 °C [13]. The metakaolin produced by flash calcination contains a higher amount of spherical particles [14]: these particles are composed of aluminosilicates whose crystallinity can vary due to the temperature gradient. When exposed to temperatures greater than 900 °C, metakaolin changes its physical form and becomes amorphous silica, resulting in a loss of pozzolanic activity as well as in reduced physical properties [13,14].

Unlike blast-furnace slag and limestone filler, metakaolin is not an industrial waste but a material that is intentionally produced. Metakaolin requires a significant amount of water during its preparation to maintain satisfactory workability, and using it alone leads to a very poor consistency and an inability to set. Therefore, it is necessary to use metakaolin in addition to a mixture. Adding up to 20% of metakaolin to slag pastes enhances the formation of an Al-substituted calcium silicate hydrate C-A-S-H gel [16,17]. The fineness of metakaolin particles is generally smaller compared to slag and PC particles and metakaolin exhibits a higher pozzolanic reactivity when used in concrete. The type of activator used affects the rheology of the paste. Therefore, a good ratio between water and precursor is required to ensure good workability [18,19].

Several studies have investigated the effect of metakaolin substitution in different alkali-activated pastes and mortars [20]. A 10% substitution has been shown to be optimal in terms of workability and compressive strength [21], while other researchers indicate that 15% or even 30% might yield better results [22]. The addition of metakaolin is particularly beneficial for both early-age strength and long-term performance because of the smaller size of the metakaolin particles compared to typical precursors. These smaller particles fill the micro-pores and improve the compactness of the paste [23,24]. Because the fineness of metakaolin is higher than that of the material it substitutes, more solution is required to increase the dissolution rate [21]. In turn, this can reduce the workability after a certain substitution rate [20,25].

Replacing 5% to 10% of slag with metakaolin results in a delayed and reduced heat flow, indicating a reduced early-age reaction rate [26]. Cements substituted with 20% metakaolin have setting times 21% shorter than that of Portland-composite cements, suggesting that metakaolin acts as a reaction accelerator or catalyst on PC due to the fine reactive particles of metakaolin [27,28,29], which is the opposite of the effect on AAMs.

A study on alkali-activated slag–fly ash paste with a substitution of slag with metakaolin revealed a reduction in autogenous shrinkage of about 24% at 7 days when the substitution rate was increased to 10% [26]. The self-desiccation was reduced when metakaolin was present in the mix due to the increased porosity and reduced chemical shrinkage. The shrinkage linked to the self-desiccation was then mitigated. In PC pastes, where up to 20% of the cement was replaced by metakaolin, a decrease in autogenous shrinkage was observed [11]. One factor contributing to this decrease is the dilution effect of metakaolin: the water-to-cement ratio or the solution-to-slag/fly ash ratio is increased when the metakaolin replacement is increased, resulting in less shrinkage [11]. Because of the fineness of metakaolin, heterogeneous nucleation is possible [11]. The particles of metakaolin can serve as a nucleation site for hydrates, which can result in higher autogenous shrinkage. However, the pozzolanic activity of metakaolin decreasing the autogenous shrinkage can become significantly higher than the effect of the heterogeneous nucleation. On the contrary, Wild et al. [30] reported increased self-desiccation because of high water consumption due to the PC hydration as well as the pozzolanic reaction of metakaolin. However, the formation of certain reaction products such as S_2_ASH_8_ in greater quantity or C_4_AH_13_ in lower amounts due to the presence of metakaolin might induce less autogenous shrinkage. Akcay et al. [31] demonstrated that the replacement of cement with metakaolin had different effects depending on the water-to-binder (W/B) ratio. At a higher W/B ratio, the autogenous shrinkage was reduced while it increased with lower W/B because of the formation of calcium hydroxide and the pozzolanic reactions of metakaolin.

Finally, no studies have been found on the effect of metakaolin on thermal strains or coefficient of thermal expansion (CTE) in AAMs, which was expected because of the already limited studies on this parameter for more simple alkali-activated pastes [7].

**Figure 1:**
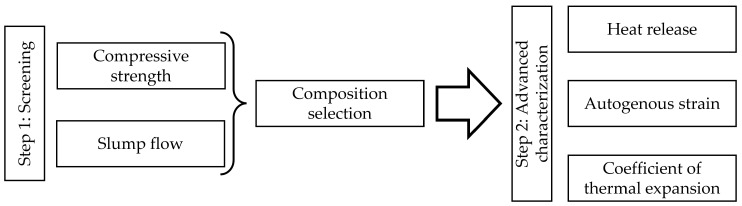
Testing campaign [12].

This study presents a two-phase investigation into the use of metakaolin as a partial replacement material and its potential role in mitigating volume changes in alkali-activated slag (AAS). To the best of the authors’ knowledge, no other paper about volume changes has investigated the effect of substitution of slag by metakaolin in compositions made with only one precursor and one activator. Most studies investigate more complex compositions made with more than one precursor. When only blast-furnace slag is used as a precursor, the activator is a mix of sodium hydroxide and sodium silicate (often). The investigation is carried out in a way that is similar to Lacante et al. [12]; see Figure 1. The first phase comprises a preliminary experimental campaign aimed at selecting suitable mixes between compositions with different solution-to-binder ratios (S/B), alkaline-activating solution concentrations, and metakaolin substitution rates, based on compressive strength and workability. Following this screening process, five mixes are selected for comprehensive evaluation in the second phase. This subsequent campaign focuses on assessing the influence of metakaolin substitution on reaction kinetics, autogenous strain, and the coefficient of thermal expansion.

The present study is preceded by previous research on early-age volume changes in AAMs. Lacante et al. [6] investigated these properties within paste made from blast-furnace slag and fly ash activated by sodium hydroxide and sodium silicate. Because of the complex behavior of these compositions, Lacante et al. [7] investigated simpler compositions made with blast-furnace slag activated by sodium hydroxide. Lacante et al. [8] went further into the analysis with an exploration of the acoustic emission activity, ultrasonic pulse velocity, and E-modulus, as well as a long-term evaluation of the autogenous strains and coefficient of thermal expansion. Because of the high strains, different mitigating possibilities were assessed. Lacante et al. [9] studied the effect of different curing temperatures (10 °C, 20 °C and 30 °C) while Lacante et al. [12] investigated the suitability of limestone filler in NaOH-activated blast-furnace slag compositions. The present study, investigating the possible mitigation effect of metakaolin in the same reference compositions, serves as the latest in this series of research studies.

## 2. Materials and Methods

### 2.1. Materials

Previous studies by Lacante et al. investigated pastes prepared with blast-furnace slag (BFS) activated with sodium hydroxide [8], which are used in the present study as the reference compositions; later, they investigated the mitigation of volume changes in some of these reference compositions by substituting blast-furnace slag with limestone filler [12]. The reference compositions in the present study are the same as in Lacante et al. [12]. The primary focus lies on the activation by sodium hydroxide (NaOH). The NaOH solutions are prepared by dissolving NaOH pellets in water at least 24 h prior to mixing. Additionally, the activation by sodium silicate (Na_2_SO_3_) is examined to compare the influence of the type of activator on volume changes in AAS at an early age.The solution of sodium silicate is commercially available 10 M Na_2_SO_3_. Because of the high concentration and the high viscosity, water is also added to the mix in a 1-to-1 mass proportion.

The chemical composition of the materials can be found in Table 1. The Blaine fineness values of the slag and the metakaolin (MK) are 4690 cm^2^/g and 33,680 cm^2^/g, respectively. The specific gravity values of the slag and the metakaolin are 2.87 g/cm^3^ and 2.50 g/cm^3^, respectively. These characteristics have been determined following the European standard EN 196-6 [32].

### 2.2. Compositions

#### 2.2.1. Reference Composition

Table 2 presents the reference compositions, where P-SXXMY is a paste (P) with S/B = X.X and Y [mol/L] is the concentration of the alkaline solution. The solution-to-binder (S/B) represents the mass ratio between the amount of solution (=alkaline solution + water if used) and the amount of binder (=slag + metakaolin if used).

The internal parameters impacting the autogenous strain development in AAM are the alkaline concentration and the solution-to-binder (S/B) ratio [33]. This translates into the effect of the water-to-cement ratio in cementitious materials [34]. The S/B ratio was set equal to 0.5 and 0.8 because lower S/B lead to compositions with poor workability [33]. On the other hand, compositions with a higher S/B ratio carry a risk of bleeding. The NaOH molar concentrations were selected equal to 2 and 8 molar. Lower concentrations induce lower reaction rates. This can lead to insufficient strength development. On the other hand, a higher concentration leads to very high reactions and temperatures. The reaction temperature as well as the solution temperature during preparation were significantly increased. Finally, the setting ages are closely related to the alkaline solution concentration [9]. Therefore, higher concentrations may lead to a fast workability loss, introducing problems during casting.

#### 2.2.2. Metakaolin Substitution

The metakaolin compositions are separated into five categories, as shown in Table 3. Categories A, B, and D are based on the reference compositions P-S08M2, P-S08M8, and P-S08NS10, respectively. Due to the high water demand of metakaolin and our aim to investigate a broader range of compositions for its impact on compressive strength and workability, two additional categories were included. Category C was added to examine a higher activator concentration of 10 M [35,36]. A fifth category E studies several distinct compositions. ME1 is based on P-S05M2 with a substitution rate of 10%. Given the high demand of water for metakaolin, the S/B = 0.6 was also investigated with two concentrations and a 20% substitution rate, as well as with 8 M concentration and a 10% substitution rate. Finally, a last composition ME4 was studied, which differs from ME3 by having an increased S/B of 0.7. For the analysis of results, the slag and metakaolin are considered as the binder.

### 2.3. Methods and Devices

All materials (blast-furnace slag, metakaolin, 2 M NaOH, 8 M NaOH, 10 M Na_2_SO_3_, and deionized water) were cured in the lab at (20 ± 2) °C for at least 24 h prior to the start of the mixing. The procedures specified in the European Standard EN 196-1:2016 [37] were followed for the preparation of the paste.

#### 2.3.1. Slump Flow

The workability of the paste was determined following the procedure outlined in ASTM C230 [38]. Two measurements of the spread diameter were taken immediately after mixing, with a precision margin of ±5 mm. The second measurement was performed orthogonally to the first one. The average of both measurements was reported with the error bars denoting the observed minimum and maximum diameters.

#### 2.3.2. Compressive Strength

Compressive strength tests were performed on 50 mm cube specimens. The paste was poured into standardized molds, vibrated to remove entrapped air, and immediately covered with plastic to prevent moisture loss. Curing was performed in a controlled environment at 20 °C. In line with previous studies [39,40], two cubes were tested per composition and per age (1, 2, and 7 days). A 600 kN Galdabini hydraulic press with a sensitivity of 1 kN was used for testing, adhering to the ASTM C109 guidelines [41]. This standard stipulates that a loading rate between 900 and 1800 N/s must be used, with this range being achieved during the first half of the expected failure load. No extra tests were performed because the variation between specimens remained below 7.6%, meeting the requirements of the ASTM C109 [41].

#### 2.3.3. Apparent Density

The apparent density was determined using the weight and dimensions of the same cubes used for the compressive strength test. The error was also assessed, revealing an average deviation overtime of 0.81% and a maximum variation of 3.53%. The densities reported in the Results section correspond to the average of all measurements because no consistent trend was identified overtime. In fact, the maximum variation over time was limited to 10.14% (average error 4.60%).

#### 2.3.4. Isothermal Calorimetry

A TAM Air isothermal calorimeter, compliant with the European standard EN 196-11:2018 [42], was used to investigate the exothermic behavior of the material at 20 °C. This instrument was equipped with eight channels operating simultaneously at the same temperature. Within each channel, two ampoule positions were available: one for the sample (weighing approximately 22.5 g) and one containing sand for an inert reference. Independent heat flow sensors were embedded at each position, reducing measured noise and improving measurement stability [43].

The heat flow typically exhibits two peaks. The first peak, related to the initial dissolution of the binder, is often missed due to the ex situ mixing process and the rapid occurrence. The second peak is associated with the formation of reaction products. The period in between is called the induction period [44].

The ex situ mixing procedure respected the protocol outlined in the European standard EN 196-1:2016 [45]. Once the ampoules were filled and sealed, they were inserted in the calorimeter within 10 min after mixing began. Two ampoules per composition were monitored.

#### 2.3.5. Autogenous Strains and Coefficient of Thermal Expansion

The methodology to investigate the autogenous strains was based on the corrugated tubes procedure described in the ASTM C1698-09 [46]. The testing device was revisited and developed at Université libre de Bruxelles (ULB) [47] to simultaneously monitor thermal strains and consequently determine the coefficient of thermal expansion (CTE). Controlled temperature cycles of ±3 °C around a curing temperature of 20 °C were applied, thanks to thermal regulation. The device was also thermally insulated to reduce any thermal exchange with the outside. As the curing temperature was 20 °C, the temperature cycle applied to the samples was as follows: 20 °C, 17 °C, 20 °C, and 23 °C. These temperatures were applied to the system for 10 min. A constant plateau at the desired temperature was maintained for 80 min. This long plateau allowed us to determine the CTE based on the measured thermal strain deduced from the simultaneously measured autogenous strain. Such small thermal variations do not impact the development and evolution of the autogenous strain nor the coefficient of thermal expansion and are great enough to induced useful thermal strains [48]. Delsaute [40] previously conducted a study to establish a testing protocol and the length of each temperature period to avoid a remaining thermal gradient by ensuring that the temperature inside the specimens reaches a constant temperature. Extra steps, called “thermal boosts”, were included at the beginning of each period to speed up the reaching and the stabilization of the internal temperature. These thermal boosts were 12 °C, 25 °C, 28 °C, and 15 °C. For example, when going from 20 °C to 17 °C, a boost of 12 °C was applied. When the temperature had to be decreased, the temperature boost was applied for 30 min to obtain the aforementioned setpoints, while 25 min sufficed when the temperature had to be increased. Because of these temperature changes, an extra sample was cast, providing the internal temperature thanks to an embedded thermocouple used during the test.

The total strain measured at time *t* ($\epsilon_{tot}\left(t\right)$ [µm/m]) was composed of the autogenous strain ($\epsilon_{auto}\left(t\right)$ [µm/m]) and the thermal strain ($\epsilon_{thermal}\left(t\right)$ [µm/m]). The latter is characterized by the CTE (α(t) [µm/m/°C]) and the temperature change (ΔT(t) [°C]), as expressed in Equation (1) [7,48]:

(1) $$\epsilon_{tot}\left(t\right)=\epsilon_{auto}\left(t\right)+\epsilon_{thermal}\left(t\right)=\epsilon_{auto}\left(t\right)+\alpha \left(t\right)\cdot \Delta T\left(t\right)$$

Decoupling the strains is necessary at a very early age. However, the autogenous strain stabilizes over short intervals of time, after a certain period, which allows for calculation of the CTE within those periods [48]. To proceed to the decoupling of the strains, a sample cured at 20 °C, without thermal variations, was determined by considering the evolution of a fictitious strain on a cubic interpolation fitted to the experimental strain at the end of the cycle interval of 20 °C. This was achieved to ensure that there was no remaining thermal gradient. By subtracting the fictitious modeled strain from the measured strain in the experiment, the thermal strain was obtained. Subsequently, the CTE evolution could be computed from these obtained thermal strains. Consequently, the experimental autogenous strains could be calculated. The autogenous strains and the CTE were computed approximately every two hours. A more detailed explanation of the decoupling of the strains is available in Delsaute and Staquet [47] and Lacante et al. [7]. Each composition was evaluated by testing two samples, along with a dummy sample for the acquisition of the temperature.

## 3. Results and Discussions

### 3.1. Preliminary Campaign

**Figure 2:**
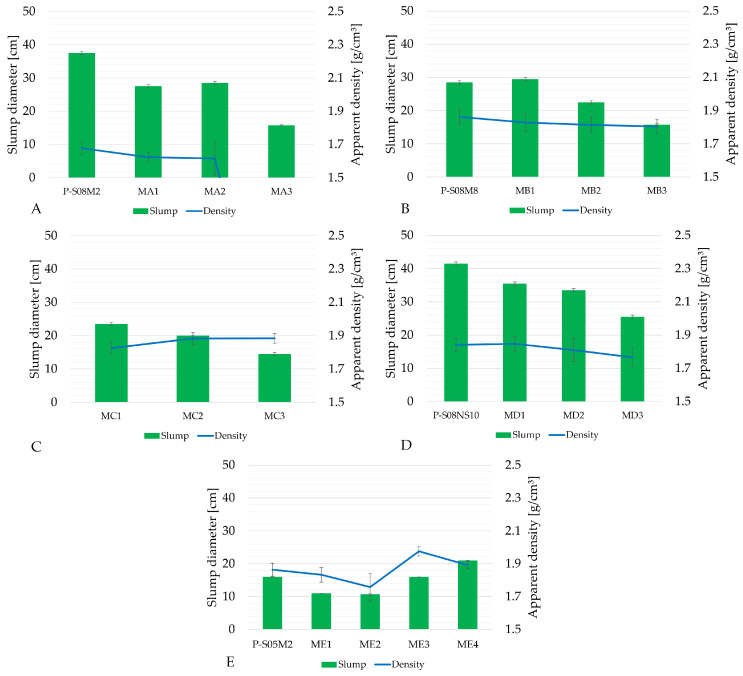
Density and slump results of AAS with metakaolin substitution: (**A**) P-S08M2 reference composition; (**B**) P-S08M8 reference composition; (**C**) P-S08M10 reference composition; (**D**) P-S08NS10 reference composition; (**E**) extra investigation. Error bars represent minimum and maximum values.

**Figure 3:**
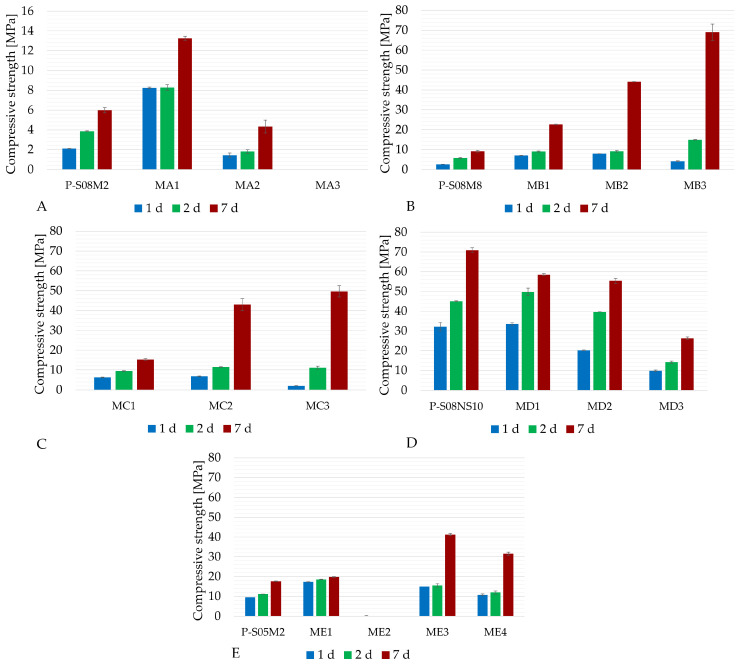
Compressive strength results of AAS with metakaolin substitution: (**A**) P-S08M2 reference composition; (**B**) P-S08M8 reference composition; (**C**) P-S08M10 compositions; (**D**) P-S08NS10 reference composition; (**E**) extra investigation. Error bars represent minimum and maximum values.

Figure 2 and Figure 3 present the apparent density and slump and compressive strength for all compositions with metakaolin substitution listed in Table 3. A comparison with the reference compositions from Table 2 is also included. Composition MA3 did not set by the time of the compressive strength test. It was still soft at the demolding and could not be tested for either apparent density or compressive strength. Although composition ME2 did set, it failed consistently during preparation for the compressive strength test: the specimen broke under the weight of the testing platform of the press (approximately 2 kN). Consequently, no force was recorded. As the amount of metakaolin increases, the water demand becomes higher. Because of this high water demand, less solution is available to react with the slag particles. Metakaolin is also less reactive than slag [49]. This results in a reduction in the production rate of the reaction products (namely C-(N)-A-S-H gels) and their quantity. This is less the case for the P-S08M8, as the alkali content is higher in this case. With the addition of metakaolin to the system, the pore refinement is delayed and the compressive strength is related to the pore structure [50]. Li et al. [50] reported that C-A-S-H gels are the dominant reaction products in alkali-activated slag. However, its quantity seems to decrease when more metakaolin is introduced into the system. Table 4 presents the percentage of compressive strength increase at 7 days compared to the reference pastes, as a function of the metakaolin substitution rate. In addition, Table 5 presents the percentage by which the compressive strength at 7 days is increased thanks to the addition of metakaolin; see Equation (2) [12].

(2) $$\frac{f_{c,x}-\left(1-x\right)\cdot f_{c,ref}}{\left(1-x\right)\cdot f_{c,ref}}$$

where $f_{c,x}$ is the compressive strength at 7 days of the composition with (*x*·100)% metakaolin substitution, $f_{c,ref}$ is the compressive strength of the reference composition at 7 days, and *x* is the substitution rate [-].

Generally, increasing the metakaolin substitution rate decreases the workability while increasing the compressive strength. However, when working with a 2 M concentration, the compressive strength decreases significantly with a 20% metakaolin substitution and the paste does not set with a 30% metakaolin substitution. This indicates that the metakaolin might not react or reacts less than slag with the alkaline solution, an effect that is also dependent on the activator concentration [26]. In addition, slag might also react less with the solution in the presence of metakaolin due to its high water demand. This will be further investigated in the in-depth experimental campaign.

In the case of the P-S08M8 composition, a 10% substitution slightly increases the workability. On the other hand, a 20% substitution results in a compressive strength four times higher than that of the reference composition [20,22].

Due to the high water demand of metakaolin, an extended investigation into the S/B = 0.5 was avoided. Instead, a study with 10 M concentration was conducted to allow direct comparison with Category B. As expected from the increase in the concentration, workability decreased, with composition MC3 being the least workable among the 0.8 S/B compositions. MB1 and MC1 had very similar compressive strengths at 1 and 2 days, but at 7 days, MB1’s compressive strength was 1.48 higher than that of MC1. MB2 and MC2 do not shown a significant difference in compressive strength. Finally, MB3’s compressive strength was consistently higher than that of MC1. The 8 M appears to be the optimal concentration for NaOH-activated pastes where slag is partially substituted with metakaolin.

When sodium silicate was used as the activator, increasing the metakaolin substitution consistently decreased the compressive strength of the pastes. However, Jithendra et al. [21] found that the addition of metakaolin could increase both the compressive strength and the workability in slag-fly ash mortars activated by sodium silicate up to a certain threshold (between 10% and 20%). It is worth noting that the concentration of the activating solution in their study was much lower (1.5–2 M). This could be related to the presence of Al_2_O_3_ and SiO_2_ in metakaolin, which introduces alumina and silica-based components in the reaction [51]. Because of the low amount of metakaolin, an initial reaction between SiO_3_^2−^ ions from the activator and the Ca^2+^ from the fly ash results in the formation of C-S-H gels [52]. Consequently, dissolved ions from the activator such as Al(OH)_4_^−^ and SiO_2_(OH)_2_^2−^ or SiO(OH)_3_^−^ possibly react with dissolved Ca^2+^ from the fly ash or Na^+^ from the sodium silicate [51]. Determined by the specific ions present, this reaction can produce C-A-S-H gel or N-A-S-H gel [53,54]. Consequently, an improvement in the compressive strength is observed. However, higher metakaolin substitution rates result in reduced formation of reaction products, while also leading to a coarser pore structure, ultimately reducing the compressive strength [50]. Tests conducted with reduced S/B ratios and metakaolin substitution reveal a significant decrease in workability, accompanied by a slight increase in compressive strength.

The main reaction product in AAS is C-A-S-H [50]. However, when metakaolin is introduced in the system, N-A-S-H will be formed [49]. C-A-S-H tends to provide early-age strength [55,56,57] while reducing workability, while N-A-S-H gels increase workability by reducing early-age strength [58,59]. The addition of metakaolin in the system increases the Al/Si ratio, which facilitates the gel conversion from C-(A)-S-H gel to C-(N)-A-S-H gel [49]. In addition, Li et al. [50] reported that after 7 days of curing, the signals associated with N-A-S-H gels become nearly invisible. However, this might not include the case where metakaolin is only in the presence of NaOH. On the other hand, silicate-activated binders exhibit higher strength compared to activation with sodium hydroxide [60]. But when metakaolin is added, the material tends to be relatively porous because of the high water demand. This could also play a role. In summary, based on the existing literature, it remains difficult to assess which differences two different activators will bring to the mix on a microstructural level and their consequences regarding the mechanical properties.

The results obtained during the selection campaign show that the use of sodium hydroxide activator leads to more favorable mechanical properties compared to sodium silicate, as the use of the latter with increasing metakaolin substitution reduced the compressive strength. The difference between 8 M and 10 M sodium hydroxide is rather negligible when the use of the 10 M NaOH solution does not decrease the compressive strength. In addition, the preparation of the 10 M NaOH solution is more challenging than that of the 8 M due to the high temperature increase when dissolving the NaOH pellets in water. In addition, it takes more time to fully dissolve. Higher concentrations also impose increased safety risks, requiring stricter handling protocols during the preparation of the solution and mixing of the material. Furthermore, using an S/B below 0.8 did not yield better results than the compositions with higher S/B ratios. Therefore, the compositions from categories C and E will not be investigated further. The most promising compositions to study in depth are the MB1, MB2, and MB3 compositions, which showed significantly increased compressive strength while maintaining satisfactory workability. Composition MA1 will also be studied further because of the high compressive strength boost and increase attributed to metakaolin, combined with good workability. Moreover, the investigation into MA1 allows us to study another NaOH concentration to compare it with MB1. Finally, MD1 will be investigated to study the impact of different activators, as it still exhibits high compressive strength and workability, despite being lower than the reference.

### 3.2. In-Depth Investigation

#### 3.2.1. Isothermal Calorimetry

To start the in-depth investigation of the selected compositions, the heat flow and cumulative heat are studied, as these follow the material’s exothermic reaction.

**Figure 4:**
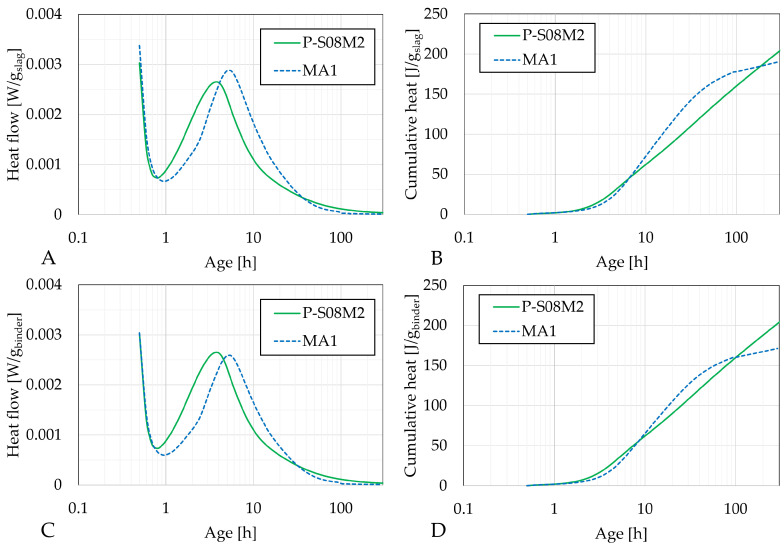
Isothermal calorimetry results for P-S08M2 and MA1: (**A**) heat flow per g of slag and (**B**) cumulative heat per g of slag; (**C**) heat flow per g of binder and (**D**) cumulative heat per g of binder (=slag+metakaolin).

**Figure 5:**
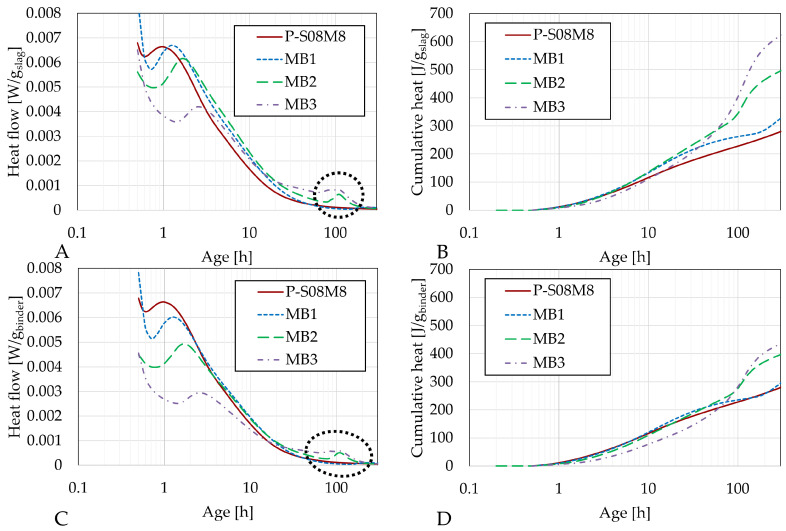
Isothermal calorimetry results for P-S08M8, MB1, MB2, and MB3: (**A**) heat flow per g of slag and (**B**) cumulative heat per g of slag; (**C**) heat flow per g of binder and (**D**) cumulative heat per g of binder (=slag+metakaolin). The black circle shows the third peak.

**Figure 6:**
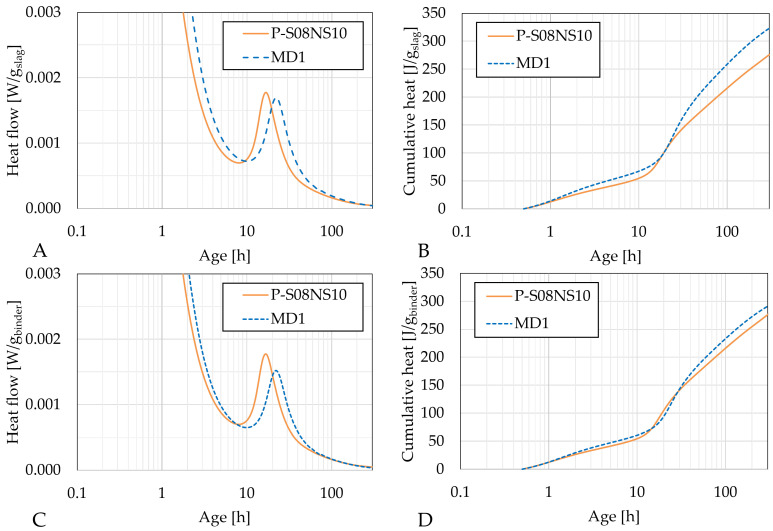
Isothermal calorimetry results for P-S08NS10 and MD1: (**A**) heat flow per g of slag and (**B**) cumulative heat per g of slag; (**C**) heat flow per g of binder and (**D**) cumulative heat per g of binder (=slag+metakaolin).

**Figure A1:**
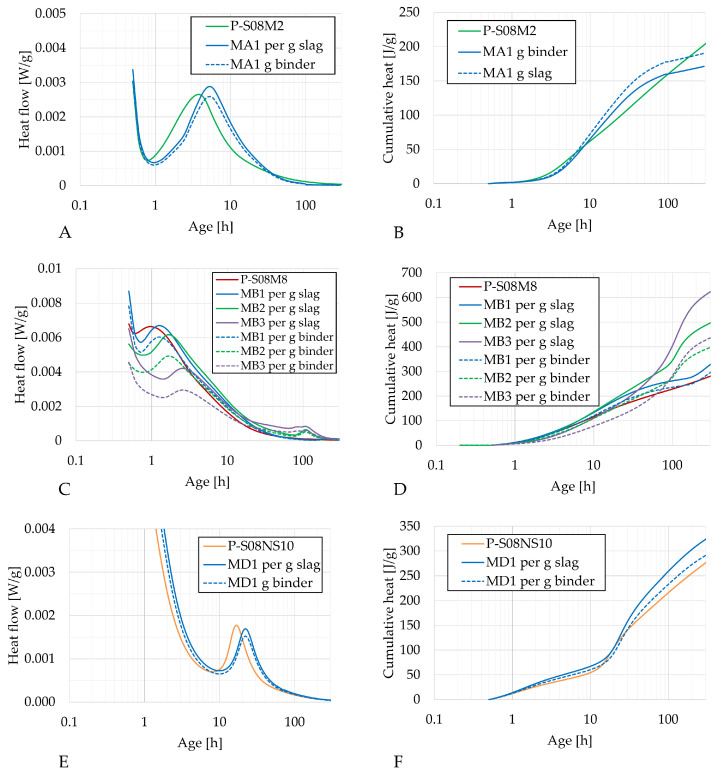
Isothermal calorimetry of all compositions per g of slag and per g of binder: (**A**) heat flow of the S08M2 reference compositions, (**B**) cumulative heat of the S08M2 reference compositions, (**C**) heat flow of the S08M8 reference compositions, (**D**) cumulative heat of the S08M8 reference compositions, (**E**) heat flow of the S08NS10 reference compositions and (**F**) cumulative heat of the S08NS10 reference compositions.

Figure 4, Figure 5 and Figure 6 present the heat flow (left) and cumulative heat (right) results of each category of selected compositions with metakaolin substitution along with their respective reference compositions: P-S08M2 and MA1; P-S08M8, MB1, MB2, and MB3; and P-S08NS10 and MD1, respectively. The results in Figure 4A,B, Figure 5A,B and Figure 6A,B are expressed per gram of slag, while those in Figure 4C,D, Figure 5C,D and Figure 6C,D are expressed per gram of binder (=slag + metakaolin). Figure A1 show a direct comparison between both normalization methods. Due to the ex situ mixing, the first peak, related to the dissolution and wetting of the solid parts [61,62,63], was not accurately monitored. Therefore, it was removed. The second peak corresponded to the formation of reaction products [33,64].

The substitution of slag with metakaolin consistently delays the appearance of the second peak, indicating a delay in the formation of the reaction products [50]. This behavior differs from that of cement pastes, where the addition of metakaolin increases the heat flow due to its catalyst nature and the higher fineness of its particles [11,65]. In terms of heat flow per gram of slag, the second peak for MA1 and MB1 is slightly higher than that of the reference composition. This might be attributed to a dilution effect [11], similar to what is observed with limestone filler substitutions [66], where the (alkaline) solution-to-slag ratio increases as the metakaolin substitution rate is increased. However, this effect is only observed with 10% substitution, suggesting that the presence of metakaolin might reduce the reactivity of slag with the activator, possibly due to the high water demand of metakaolin [67].

Interestingly, a third peak is observed for MB1 (very small), MB2, and MB3 between 70 and 200 h. This corresponds to a further increase in the cumulative heat at a later age and might indicate that the presence of metakaolin in compositions activated with 8 M NaOH triggers a delayed reaction. This late reaction could also explain the high compressive strength at 7 days in the B category compared to the reference composition, while categories A and C have similar or even reduced compressive strengths. Notably, the lower the second peak is, the higher the third peak is. This phenomenon is not observed for MA1 or MD1. A 10% substitution leads to very similar heat flow and cumulative heat compared to the reference compositions. A 30% substitution results in a second peak that is nearly half of the magnitude of the reference (in gram per binder). In addition, the cumulative heat remains lower until the third peak appears, after which it surpasses that of the reference composition. Metakaolin-based geopolymers have been investigated in the literature. Li et al. [68] investigated their chemical shrinkage and found three stages in the evolution. The first stage corresponds to the chemical deformation due to the dissolution of the precursors [69]. This is followed by a chemical expansion, associated with the formation of aluminum-rich phases (e.g., nano-zeolites) [70]. Lastly, the chemical shrinkage after 50 h was associated with the polymerization and the reorganization of the previously formed entities (formation of silicon-rich gels [69]). This last phenomenon could explain the third heat flow peak.

When sodium silicate is used as the activator, the substitution of slag by metakaolin does not significantly impact the cumulative heat. Per gram of binder, the only noticeable effect is a slight delay in the reaction rate. Per gram of slag, the cumulative heat is higher, but the overall behavior of the curve remains unchanged, in contrast to the results observed with NaOH activation.

The impact of the slag substitution by metakaolin is assessed in Table 6. For P-S08M2, the effect is positive in the beginning but becomes negative over time. Conversely, for both P-S08M8 and P-S08NS10, the inclusion of metakaolin in the matrix contributes negatively in the beginning but becomes positive over time. The higher the substitution rate, the higher the impact is.

Next, the ultimate heat was computed using two different methods, based on the cumulative heat [12]. The first method involves fitting a polynomial equation to the cumulative heat as a function of the inverse of the square root of the age. A second-degree polynomial equation is fitted over the age interval [25 h, 314 h], as described in Equation (3). In line with the approach taken for the isothermal calorimetry results, the ultimate heat has been computed both per gram of slag and per gram of binder. The fitting parameters *a*, *b*, and $Q_{\infty ,1}$, as well as the fitting error, are shown in Table A1.

(3) $$Q\left(\frac{1}{\sqrt{t}}\right)=a\cdot {\left(\frac{1}{\sqrt{t}}\right)}^{2}-b\cdot \frac{1}{\sqrt{t}}+Q_{\infty ,1}$$

The second method is a variation of the Freiesleben Hansen and Pedersen model, involving an exponential fitting adapted for the data’s multi-curvature nature [71,72,73,74]; see Equation (4), where *a* and τ are material parameters for the curvature and the curve’s intercept, respectively, and $Q_{1}+Q_{2}=Q_{\infty ,2}$.

(4) $$Q\left(t\right)=Q_{1}\cdot exp\left(-{\left(\frac{\tau_{1}}{t}\right)}^{a_{1}}\right)+Q_{2}\cdot exp\left(-{\left(\frac{\tau_{2}}{t}\right)}^{a_{2}}\right)$$

The age fitting interval for this second method is [0.5 h, 314 h], while the sum of squares was minimized during the fitting. All the corresponding fitting parameters can be found in Table 7 and Table A1.

The two methods result in different ultimate heat values. For composition MA1, an increase in metakaolin substitution consistently leads to a reduction in the ultimate heat, regardless of the method used. In contrast, for composition MD1, the trend depends on the method: the polynomial fitting results in a higher ultimate heat, while the exponential fitting indicates a lower value. In the case of compositions from Category B, the higher the substitution rate, the higher the ultimate heat is, except for MB2 with the exponential fitting. It is worth noting that MB1 exhibits the highest fitting error due to the late appearance of the third peak, complicating the curve fitting. Therefore, its ultimate heat might indeed be lower than predicted. The results normalized per gram of binder are consistently lower than those normalized per gram of slag (except for MB1).

Finally, in line with previous findings on limestone filler substitution [12], compositions activated with sodium silicate show significantly high ultimate heat values when fitted with the exponential method. This suggests that the exponential method might not be the most suitable approach for such materials, likely due to their disproportionate multi-curvature behavior compared to compositions activated with sodium hydroxide.

Lastly, the degree of reaction (DOR) can be computed thanks to the ultimate heat by using Equation (5), where *i* is 1 (polynomial method) or 2 (exponential method). This facilitates the comparison of the autogenous strain and the coefficient of thermal expansion across different compositions. Furthermore, it provides an opportunity to evaluate the influence of the two computational methods to determine this parameter.

(5) $$DOR_{i,slag\;or\;binder}\left(t\right)=\frac{Q(t)}{Q_{\infty ,i,slag\;or\;binder}}$$

#### 3.2.2. Autogenous Strain

**Figure 7:**
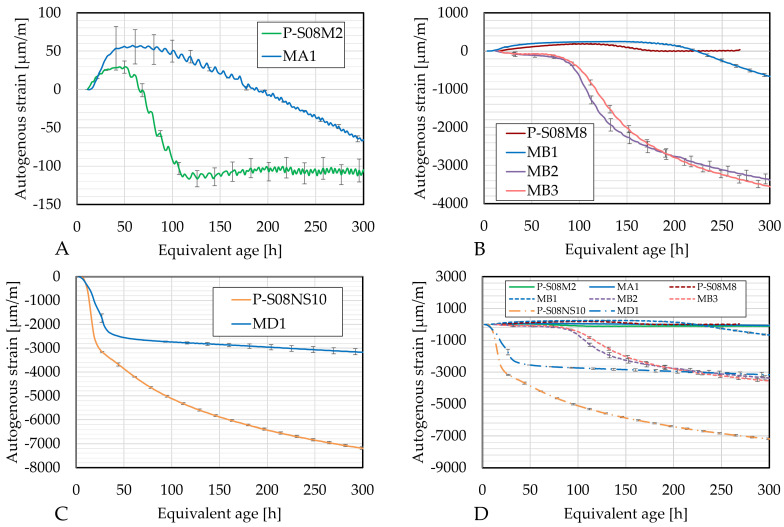
Autogenous strain as function of age: (**A**) P-S08M2 reference composition; (**B**) P-S08M8 reference composition; (**C**) P-S08NS10 reference composition; (**D**) summary of all compositions. Error bars represent minimum and maximum values.

The autogenous strain as a function of the age of each composition as well as the respective reference composition is presented in Figure 7. The knee-point method has been used to initialize the autogenous strain curves: the initialization point corresponds to the time when the autogenous strain rate reaches zero [75]. If that does not occur, the initialization age corresponds to the time when the strain rate reaches its maximum [7]. The initialization ages and the corresponding DOR are presented in Table 8. Notably, for several compositions, the initialization DOR remains the same regardless of whether it is computed based on the ultimate heat per gram of binder or per gram of slag.

Several compositions undergo (apparent) swelling at the beginning of the data. During the reaction, reaction products (such as hydrotalcite group minerals) are formed [76]. These might be at the origin of the swelling, similar to PC pastes where ettringite is responsible for the swelling [10]. The autogenous strain and the internal relative humidity (IRH) are closely linked together: as the internal relative humidity increases, the autogenous strain increases as well, resulting in swelling [77]. The Kelvin radius of the pores increases as the internal relative humidity increases. Consequently, the reduction in the pressure induces swelling. Finally, swelling can also be attributed to the reabsorption of the alkaline solution leading to a volume increase [10].

The initial swelling phase is followed by autogenous shrinkage. Alkali-activated materials typically develop a dense pore structure, which leads to higher surface tension in the pore solution. As a result, the capillary pressure increases due to the elevated degree of saturation [48]. In addition, AAMs exhibit greater deformability, as their C-A-S-H gel phase is highly viscoelastic [76]. Shrinkage is also increased by the progressive polycondensation of gel units during the formation of the solid network. This process reduces the interparticle distance, contributing to shrinkage [78]. Finally, force imbalances within the system further increase shrinkage. As the ion concentration in the pore solution decreases over time, repulsive steric-hydration forces diminish, while attractive forces between gel particles remain the same, leading to shrinkage [79].

Only MA1 and MB1 exhibit swelling, suggesting that substituting 10% of slag with metakaolin might enhance swelling—caused, in alkali-activated slag, by the expansion of the reaction products [76], the increase in IRH [77], and the reabsorption of the solution [10]. In addition, the solution-to-slag ratio increases with the metakaolin substitution increase, which could contribute to swelling. The swelling occurring in these materials might also be caused by the expansive process occurring with the N-A-S-H gels formation and thus the intrinsic gel structure evolution, which has been observed in systems made from metakaolin activated with sodium silicate and sodium hydroxide (AAMK) [80]. The swelling is followed by shrinkage, which may be related to two mechanisms observed in AAMK [80]. The expansive process related to the N-A-S-H gels slows down over time, while the already formed gels start ageing and induce shrinkage, which may also be linked to a molecular-scale restructuring. The mitigating effect of metakaolin can also be related to the reduced pore refinement [50]. For both compositions, no plateau is observed, as it is the case for their reference compositions. Although the swelling is magnified, the subsequent shrinkage rate remains constant for the rest of the testing time. However, the rate of shrinkage is reduced compared to the reference composition.

Compositions MB2 and MB3 exhibit similar behavior. Both show some initial shrinkage. Around 76 h, the shrinkage increases significantly. This moment coincides with the onset of the third peak in the heat flow data, suggesting that the formation of reaction products might induce more shrinkage. Interestingly, the autogenous shrinkage for both these compositions is very similar, with only a 5.22% difference at 300 h, despite the steeper increase in shrinkage of MB2 and the observed differences in heat flow, cumulative heat, compressive strength, and workability.

Composition MD1 is the only composition with a significant reduction in autogenous shrinkage: 51% at 24 h, 53% at 168%, and 56% at 300 h. This shrinkage reduction is relatively higher than what Li et al. [50] found for slag activated by sodium hydroxide and sodium silicate: 44% at 24 h and 38% at 168 h for a 10% substitution. On the other hand, a 20% substitution with metakaolin led to a shrinkage reduction of more than 60% at 24 h, and 50% at 168 h of age. Similarly, previous studies have generally found that the addition of metakaolin to alkali-activated pastes results in decreased autogenous shrinkage [11,26]. However, the materials studied were cement paste with metakaolin [11], slag and fly ash activated by sodium hydroxide and sodium silicate [26], and slag activated by sodium hydroxide and sodium silicate [50]—with no similar research specifically on slag activated by only sodium hydroxide. This suggests that the effect of metakaolin on shrinkage is highly dependent on the used activator. In blended compositions such as slag and fly ash activated with sodium hydroxide and sodium silicate, the shrinkage is reduced with the effect of metakaolin in the presence of sodium silicate, which seems to be more significant than the shrinkage-inducing effect of metakaolin activated with sodium hydroxide. Because of the high shrinkage induced by metakaolin in the presence of sodium hydroxide, it is expected that the shrinkage reduction induced by the metakaolin in the presence of only sodium silicate would be higher than the shrinkage reduction observed in systems composed of blast-furnace slag activated by both activators, such as in Li et al. [50]. This expectation is correct. Furthermore, the individual contribution of fly ash in these blended systems, as opposed to slag alone, remains uninvestigated.

**Figure 8:**
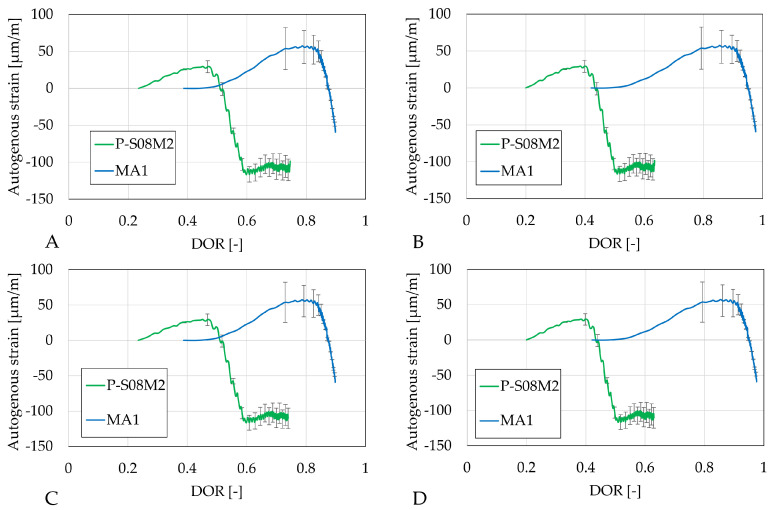
Autogenous strain of S08M2 reference compositions as degree of reaction: (**A**) calculated with Q_*∞*,1,slag_; (**B**) calculated with Q_*∞*,2,slag_; (**C**) calculated with Q_*∞*,1,binder_; (**D**) calculated with Q_*∞*,2,binder_. Error bars represent minimum and maximum values.

The autogenous strain as a function of the different DORs is presented in Figure 8. The same ultimate heat computation methods (A and C, and B and D) yield similar results compared to the computation per gram of slag or binder. Substitution by metakaolin induces a delay in evolution, particularly on the shrinkage (which only occurs after DOR = 0.8).

**Figure 9:**
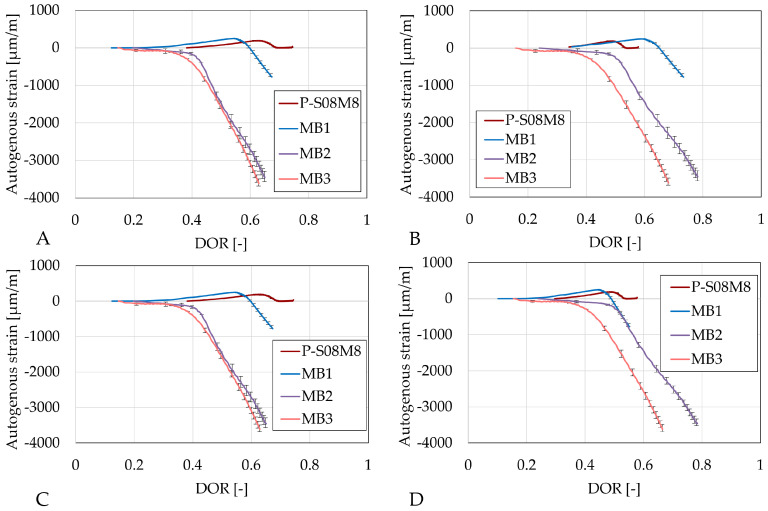
Autogenous strain of S08M8 reference compositions as degree of reaction: (**A**) calculated with Q_*∞*,1,slag_; (**B**) calculated with Q_*∞*,2,slag_; (**C**) calculated with Q_*∞*,1,binder_; (**D**) calculated with Q_*∞*,2,binder_. Error bars represent minimum and maximum values.

The autogenous strains of Category B (P-S08M8, MB1, MB2, and MB3) as a function of the different DORs are presented in Figure 8. The same ultimate heat computation methods (A and C, and B and D) yield again similar results compared to the computation per gram of slag or binder, except for MB1 as a function of DOR computed with Q_*∞*,2,slag_ and Q_*∞*,2,binder_ (see Figure 9B,D). Depending on the computation method, the onset of shrinkage increase occurs faster with respect to the reference composition. Except for Figure 9B, the increase in metakaolin substitution accelerates the appearance of shrinkage.

For all Category B compositions with metakaolin, the relation between the shrinkage and the DOR is linear.

**Figure 10:**
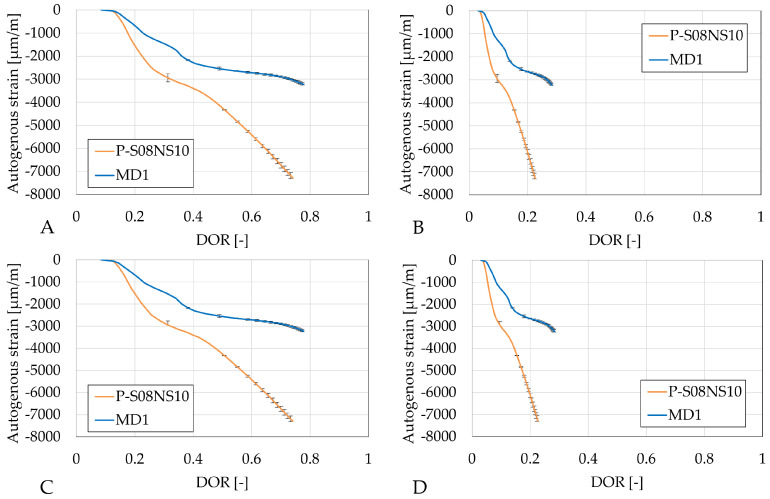
Autogenous strain of S08NS10 reference compositions as degree of reaction: (**A**) calculated with Q_*∞*,1,slag_; (**B**) calculated with Q_*∞*,2,slag_; (**C**) calculated with Q_*∞*,1,binder_; (**D**) calculated with Q_*∞*,2,binder_. Error bars represent minimum and maximum values.

Figure 10 reveals again very similar results between different computation methods for DOR (A and C, and B and D). The slag substitution by metakaolin significantly reduces the autogenous shrinkage.

#### 3.2.3. Coefficient of Thermal Expansion

**Figure 11:**
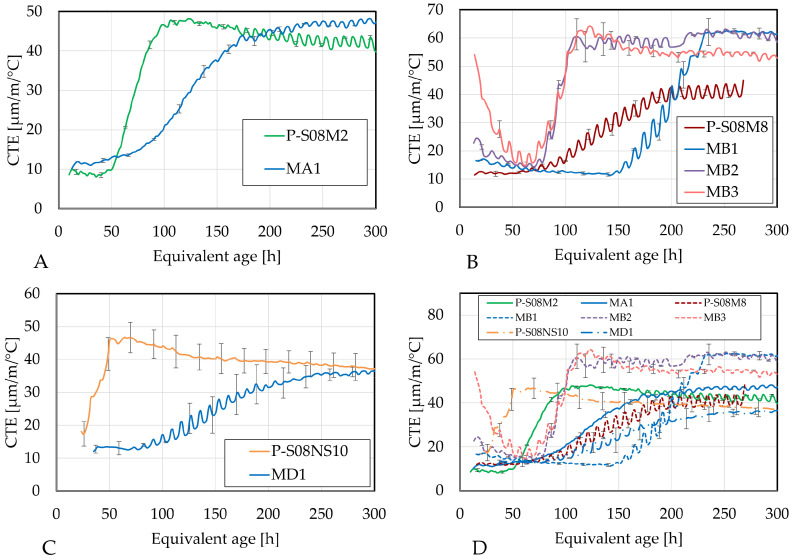
Coefficient of thermal expansion as function of the age: (**A**) P-S08M2 reference composition, (**B**) P-S08M8 reference composition, (**C**) P-S08NS10 reference composition, (**D**) summary of all compositions. Error bars represent the minimum and maximum values.

As alkali-activated materials generally exhibit high coefficients of thermal expansion (CTEs) [6,7], the effect of metakaolin on the CTE was also investigated. Figure 11 presents the CTE for each composition as well as each reference composition, as a function of age.

Compositions MA1 and MB1 exhibit a delayed evolution in their CTE as well as relatively higher initial and final CTE values compared to the CTEs of their respective compositions. This phenomenon might be related to the higher solution-to-slag ratio in these compositions. A higher solution-to-slag ratio delays the evolution of the CTE but also increases the CTE once the evolution starts [8] due to the presence of more liquid in the material and the formation of more pores resulting in higher pressure when the temperature varies [48] and because of the increase in the CTE of sodium hydroxide as the concentration increases [81]. Yeon et al. [82] have indicated that the meniscus’ curvature at the interface between vapor and liquid water is affected by temperature variations in Portland cement. The capillary stress as well as the pore fluid pressure influence this, too. The CTE can be impacted as these are key mechanisms defining volume changes. Negative pore pressure in the fluid resulting from temperature changes results in water molecules’ contraction at the meniscus. In addition, when the temperature is increased, the internal relative humidity is also increased because of the pore water expansion. Consequently, the waterfront moves outward because the meniscus radius is increased. Through the Kelvin–Laplace equation, the interfacial tension between the water and the air is reduced with a temperature increase [83]. Moreover, Li et al. [50] observed delayed pore refinement when metakaolin was used as a partial substitution for slag (activated by sodium hydroxide and sodium silicate). This can induce reduced thermal strain. When the pore structure is less refined, the pore pressure and the capillary pressure are already reduced. In addition, the less-refined pore structure leads to an increase in micropore volume and a decrease in mesopore volume [84]. Consequently, the effect of the temperature changes on the material might be reduced due to the presence of more mesopores. However, afterwards, a refinement of the pore structure can be expected, as it is only delayed [50]. Therefore, the CTE still increases after some time.

As the substitution of slag by metakaolin is increased, the evolution of the CTE is sped up, reaching a CTE of about 60 µm/m/°C after 110 h. Until this age, the compositions with 20% and 30% metakaolin substitution rates yield a relatively similar increase, with the increase in initially measured values powered by the increase in the metakaolin substitution rate. After this age, the composition with 30% substitution rate slightly decreases, reaching a CTE of 52.93 µm/m/°C at 300 h. In comparison, MB2 maintains a CTE around 60.54 µm/m/°C, and MB1’s CTE is slightly higher at 61.36 µm/m/°C. The rapid increase in CTE of MB2 and MB3 starts around 75 h, which corresponds to the onset of the third peak in the heat flow. This suggests that the increase in CTE is likely linked to the same phenomena that produce the increase in autogenous shrinkage.

Composition MD1 is also slowed down compared to its reference composition. However, this time, it does not surpass the CTE of P-S08NS10 before 300 h. Around that time, the CTE of reference composition is decreasing while MD1 still increases, suggesting that the latter will surpass the former at some moment. However, both compositions exhibit a decrease in their rate over time.

The consistent delay in the evolution of CTE when 10% of the slag is substituted is a positive aspect, as it indicates that the material will have reduced cracking potential at an early age, provided that the tensile strength evolution is not delayed. Based on the results for compressive strength, the strength development does not seem to be impacted as much. However, the higher stabilization of CTE might increase the cracking potential at later age. Although the 30% substitution rate accelerates the CTE development compared to the reference composition P-S08M8, it still progresses slower than that of P-S08M2. Depending on tensile strength and elastic modulus evolution, it might still be more beneficial to use MB3. In addition, the CTE of MB3 decreases again, which does not seem to be the case for the other metakaolin compositions. Regarding the flexural strength of these types of materials, Dai et al. [85] observed a decrease in flexural strength with an increase in metakaolin substitution. They also reported a slight reduction in compressive strength when the metakaolin substitution of slag was increased from 10% to 20% with 4.2 M NaOH activation. However, with a 5.8 M NaOH solution, metakaolin substitution from 10% to 20% still resulted in a reduction in flexural strength, though the decrease was much less pronounced compared to the former case, while an increase in compressive strength was also noted.

**Figure 12:**
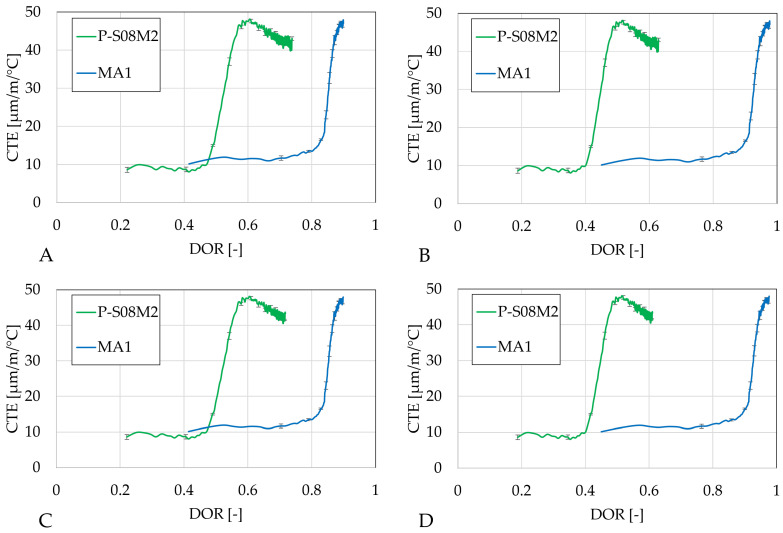
Coefficient of thermal expansion of S08M2 reference compositions as a function of the degree of reaction: (**A**) calculated with Q_*∞*,1,slag_; (**B**) calculated with Q_*∞*,2,slag_; (**C**) calculated with Q_*∞*,1,binder_; (**D**) calculated with Q_*∞*,2,binder_. Error bars represent the minimum and maximum values.

In terms of DOR, the evolution of the CTE is delayed when the metakaolin substitution rate is increased, regardless the computing method of the ultimate heat; see Figure 12.

**Figure 13:**
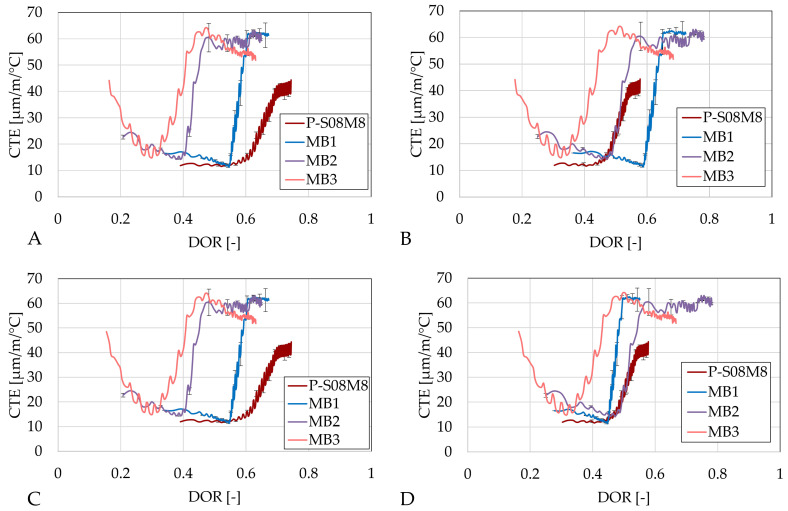
Coefficient of thermal expansion of S08M8 reference compositions as a function of the degree of reaction: (**A**) calculated with Q_*∞*,1,slag_, (**B**) calculated with Q_*∞*,2,slag_, (**C**) calculated with Q_*∞*,1,binder_, and (**D**) calculated with Q_*∞*,2,binder_. Error bars represent the minimum and maximum values.

Unlike other categories, the analysis of Category B in terms of DOR depends on the method used to compute the ultimate heat; see Figure 13. While MB3 consistently increases earlier in terms of DOR compared to the other compositions in this category, the same can not be concluded for MB1 and MB2. When the DOR is computed based on ultimate heat calculated with the polynomial method, the development of the CTE occurs sooner but the rate is slightly reduced when the metakaolin substitution is increased. However, the rate remains higher than that of the reference composition. When the DOR is computed based on the ultimate heat determined with the exponential method, the analysis depends on whether it is computed per gram of slag or per gram of binder. With Q_*∞*,2,slag_, the development of the CTE of MB1 is delayed, while MB2’s CTE is similar to that of the reference composition. For MB3, the CTE evolution occurs sooner. On the other hand, when Q_*∞*,2,binder_ is used, MB2’s CTE development is again similar to that of the reference composition. However, MB1 shows an earlier increase in CTE, and MB3’s CTE increases even sooner in terms of DOR.

**Figure 14:**
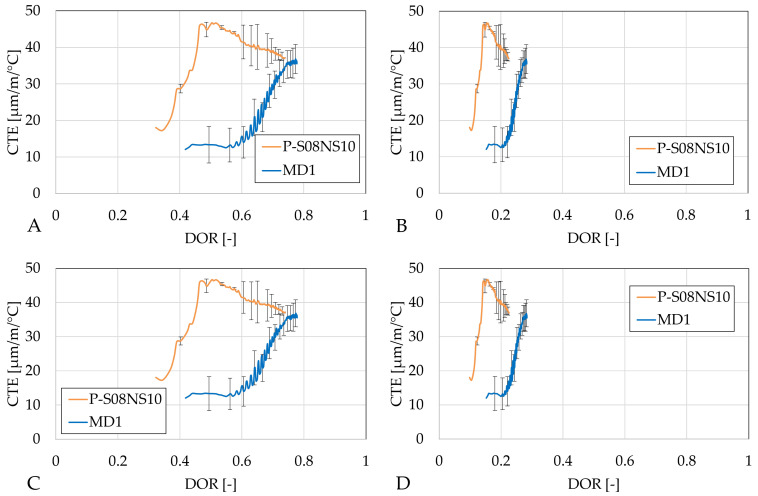
Coefficient of thermal expansion of S08NS10 reference compositions as a function of the degree of reaction: (**A**) calculated with Q_*∞*,1,slag_, (**B**) calculated with Q_*∞*,2,slag_, (**C**) calculated with Q_*∞*,1,binder_, and (**D**) calculated with Q_*∞*,2,binder_. Error bars represent the minimum and maximum values.

When sodium silicate is used as the activator, the development of the CTE is also slowed down; see Figure 14. In addition, the development of the CTE is altered when the metakaolin substitution rate is increased from 0 to 10%. Nevertheless, both CTEs appear to converge towards the same final CTE value.

## 4. Conclusions and Perspectives

This study aimed to assess the mitigating potential of metakaolin on high volume changes in alkali-activated materials. The following conclusions can be drawn from the different results and discussions.

1. A preliminary study investigated different metakaolin substitution rates in terms of compressive strength and workability. Five compositions were selected and examined in detail. MA1 and MB1 exhibited a compressive strength increase of 122% and 146%, respectively, at 7 days for a 10% substitution rate. In contrast, MD1 showed a decrease of 28%, already indicating the important role of the activator. In addition, MB2 and MB3 showed a remarkable 380 and 650% increase in the compressive strength for a 20% and 30% metakaolin substitution rate, compared to the reference composition. However, the workability decreased as the metakaolin substitution rate increased.
2. Isothermal calorimetry results indicated that metakaolin does affect the heat release. At a 10% substitution rate, compositions activated with 2 M NaOH and 10 M sodium silicate had a slightly delayed second heat flow peak. In contrast, compositions activated with 8 M NaOH exhibited the appearance of a third peak. For the latter, as the substitution rate was increased, the second peak was decreased and delayed while the third peak increased. The reduction in and delay of the heat flow evolution might be related to the dilution effect caused by the presence of metakaolin in the mix. After this, aluminum-rich products are formed. Finally, the third peak might be related to the polymerization and reorganization of the previously formed entities.
3. The autogenous swelling increased with a 10% metakaolin substitution. Later on, the subsequent shrinkage appeared at a constant rate. Compositions MB2 and MB3 exhibited very similar autogenous strain developments: no apparent swelling was observed and the autogenous shrinkage was significantly increased. The start of the increase in shrinkage rate coincides with the appearance of the third peak in the heat flow. At 300 h, only a 5% difference was observed between the two compositions. The effect of metakaolin on the autogenous strain development is strongly dependent on the activator: while a sodium hydroxide activation altered the behavior of the autogenous strain development, the autogenous train of sodium silicate activated composition was significantly reduced. This explains the mitigating effect of metakaolin observed in the literature with precursors activated with sodium hydroxide and sodium silicate.
4. The effect of the metakaolin substitution on the CTE was also dependent on the activator and the substitution rate. A 10% substitution rate caused a delay in the development of the CTE, possibly due to the dilution effect. In addition, these compositions stabilized higher than the reference composition when activated by sodium hydroxide. Substitution rates of 20% (MB2) and 30% (MB3) in the reference composition P-S08M8 led to a faster CTE increase with respect to age. However, MB3 slightly decreased again, while MB2 remained constant for the rest of the test. This fast increase started around the time the third peak in the heat flow appeared.

The tensile strength of the studied materials as well as the evolution of the E-modulus should be investigated to evaluate their cracking potential. Given that MB2 and MB3 exhibit similar autogenous and thermal strains, it might be interesting to determine whether their tensile strength and E-modulus differ as significantly as their compressive strength. Under restrained conditions, this will be crucial in determining if the material is prone to cracking.

In addition, further investigation into the third peak of the heat flow is necessary to identify which reaction products are made or what initiates this late reaction, which is not seen in other compositions. Furthermore, inspection of the microstructure with SEM or XRD as well as of the pore structure with MIP is an important aspect to elaborate and to support the macroscale observations and conclusions.

## Figures and Tables

**Table 1:** Chemical composition [%] of the materials in mass percent.

Material	SiO_2_	Al_2_O_3_	Fe_2_O_3_	CaO	K_2_O	MgO	TiO_2_	SO_3_	Na_2_O	BaO	MnO	SrO	V_2_O_5_	P_2_O_5_	LOI
BFS	34.20	12.86	0.35	39.95	0.62	7.91	1.13	1.88	0.50	0.13	0.30	/	/	/	/
MK	51.04	45.66	0.57	0.06	0.14	0.16	0.85	0.03	0.30	/	/	0.004	0.03	0.12	0.98

**Table 2:** Reference compositions, where P-SXXMY represents a paste (P) with an S/B of X.X and an alkaline solution concentration of Y mol/L [12] (partially).

Compositions	S/B Ratio [-]	Alkaline Solution	Concentration [mol/L]	Water/Alkaline Solution [-]
P-S05M2	0.5	NaOH	2	0
P-S08M2 P-S08M8	0.8	NaOH	8	0
P-S08NS10	0.8	Na_2_SiO_3_	10	1/1

**Table 3:** Metakaolin compositions, where MKZZSXXMY represents a paste with an S/B of X.X, an alkaline solution concentration of Y mol/L, and a substitution rate of ZZ%.

ID	Compositions	S/B Ratio [-]	Metakaolin Ratio [%]	Alkaline Solution	Concentration [mol/L]	Water/Alkaline Solution [-]
MA1	MK10S08M2		10			
MA2	MK20S08M2	0.8	20	NaOH	2	0
MA3	MK30S08M2		30			
MB1	MK10S08M8		10			
MB2	MK20S08M8	0.8	20	NaOH	8	0
MB3	MK30S08M8		30			
MC1	MK10S08M10		10			
MC2	MK20S08M10	0.8	20	NaOH	10	0
MC3	MK30S08M10		30			
MD1	MK10S08NS10		10			
MD2	MK20S08NS10	0.8	20	Na_2_SiO_3_	10	1/1
MD3	MK30S08NS10		30			
ME1	MK10S05M2	0.5	10		2	0
ME2	MK20S06M2	0.6	20	NaOH		0
ME3	MK10S06M8	0.6	10		8	0
ME4	MK10S07M8	0.7	10			0

**Table 4:** Compressive strength increase [%] at 7 days as function of metakaolin substitution rate with respect to reference composition.

MK Substitution	P-S08M2	P-S08M8	P-S08NS10	P-S05M2
10%	121.8	146.2	−17.4	12.5
20%	−27.5	379.5	−21.8	/
30%	/	649.8	−62.8	/

**Table 5:** Compressive strength boost [%] at 7 days as function of substitution rate with respect to reference composition, computed based on Equation (2).

MK Substitution	P-S08M2	P-S08M8	P-S08NS10	P-S05M2
10%	146.4	173.6	−8.2	25.0
20%	−9.4	499.4	−2.3	/
30%	/	971.1	−46.9	/

**Table 6:** Magnification impact [%] of the substitution with metakaolin on the cumulative heat per gram of binder at different ages.

Reference Composition	MK Substitution	24 h	168 h	300 h
P-S08M2	10%	17.3	−8.6	−15.4
P-S08M8	10%	−35.9	−11.6	5.2
	20%	−39.6	27.7	41.8
	30%	−54.7	37.7	55.6
P-S08NS10	10%	−5.7	7.3	5.5

**Table 7:** Summary of the ultimate heat estimations of the MK compositions [J/g].

		P-S08M2	MA1	P-S08M8	MB1	MB2	MB3	P-S08NS10	MD1
Per of g of slag	Q_*∞*,1,slag_	278.59	211.65	368.35	494.60	770.22	993.76	376.00	421.18
	Q_*∞*,2,slag_	327.32	194.77	474.26	459.36	639.73	917.08	1233.22	1155.64
Per of g of binder	Q_*∞*,1,binder_	278.59	190.49	368.35	445.05	616.18	695.63	376.00	379.06
	Q_*∞*,2,binder_	327.32	175.29	474.26	543.44	511.79	658.58	1233.22	1040.07

**Table 8:** Initialization time for autogenous strain for each composition.

Composition	Time Zero [h]	DOR_1,slag_ [-]	DOR_2,slag_ [-]	DOR_1,binder_ [-]	DOR_2,binder_ [-]
P-S08M2	10.89	0.235	0.200	0.235	0.200
MA1	11.55	0.387	0.421	0.387	0.421
P-S08M8	15.08	0.381	0.296	0.381	0.296
MB1	3.48	0.124	0.134	0.124	0.102
MB2	12.09	0.198	0.239	0.198	0.239
MB3	15.70	0.146	0.158	0.146	0.154
P-S08NS10	5.78	0.116	0.035	0.116	0.035
MD1	2.25	0.084	0.031	0.084	0.031

## Data Availability

The original contributions presented in this study are included in the article. Further inquiries can be directed to the corresponding author.

## Abbreviations

The following abbreviations are used in this manuscript:

AAMs	Alkali-activated materials
AAS	Alkali-activated slag
CTE	Coefficient of thermal expansion
DOR	Degree of reaction
M	Concentration in mol/L
MK	Metakaolin
PC	Portland cement
q	Heat flow
Q	Cumulative heat
S/B	Solution-to-binder mass ratio

## Appendix A

**Table A1:** Ultimate heat results.

			P-S08M2	MA1	P-S08M8	MB1	MB2	MB3	P-S08NS10	MD1
Per of g of slag	Q_*∞*,1,slag_	a	3071.20	−345.42	4283.10	15,437.00	11,693.00	10,684.00	3762.60	2129.40
		b	−1490.30	−325.90	−1823.60	−3935.10	−5250.30	−6792.70	−1972.10	−1825.20
		c = Q_*∞*,1,slag_	278.59	211.65	368.35	494.60	770.22	993.76	376.00	421.18
		R^2^	0.9990	0.9936	0.9958	0.9214	0.9417	0.9856	0.9994	0.9994
Per of g of slag	Q_*∞*,2,slag_	Q1	31.96	46.06	288.94	354.13	541.80	189.25	73.88	102.91
		τ _1_	4.57	4.48	8.36	9.48	20.97	98.62	20.84	27.07
		a_1_	1.60	0.99	0.55	0.54	0.44	3.22	2.11	2.14
		Q_2_	295.36	148.71	185.32	105.23	97.93	727.83	1159.34	1052.72
		τ _2_	62.94	12.42	816.50	98,887.57	111.95	51.62	9085.82	4229.70
		a_2_	0.39	1.07	0.62	0.64	4.52	0.39	0.16	0.17
		error	31.50	213.87	46.82	143,420.47	310.17	5605.96	1035.46	1593.49
		Q_*∞*,2,slag_	327.32	194.77	474.26	459.36	639.73	917.08	1233.22	1155.64
Per of g of binder	Q_*∞*,2,binder_	a	3071.20	−310.88	4283.10	13,893.00	9354.60	7478.50	3762.60	1916.50
		b	−1490.30	−293.31	−1823.60	−3541.60	−4200.20	−4754.90	−1972.10	−1642.70
		c = Q_*∞*,1,binder_	278.59	190.49	368.35	445.05	616.18	695.63	376.00	379.06
		R^2^	0.9990	0.9936	0.9958	0.9417	0.9817	0.9856	0.9994	0.9994
Per of g of binder	Q_*∞*,2,binder_	Q1	31.96	133.84	288.94	137.78	433.44	530.19	73.88	92.62
		τ _1_	4.57	12.42	8.36	5.05	20.97	56.83	20.84	27.07
		a_1_	1.60	1.07	0.55	1.06	0.44	0.38	2.11	2.14
		Q_2_	295.36	41.45	185.32	405.66	78.34	128.39	1159.34	947.45
		τ _2_	62.94	4.48	816.50	373.21	111.95	98.59	9085.82	4229.70
		a_2_	0.39	0.99	0.62	0.23	4.52	3.31	0.16	0.17
		error	31.50	173.24	46.82	12,458.33	198.51	2756.02	1035.46	1290.73
		Q_*∞*,2,binder_	327.32	175.29	474.26	543.44	511.79	658.58	1233.22	1040.07
